# Neural Correlates of Transient Mal de Debarquement Syndrome: Activation of Prefrontal and Deactivation of Cerebellar Networks Correlate With Neuropsychological Assessment

**DOI:** 10.3389/fneur.2020.00585

**Published:** 2020-06-30

**Authors:** Seung-Ho Jeon, Yeong-Hun Park, Sun-Young Oh, Jin-Ju Kang, Yeon-Hee Han, Hwan-Jeong Jeong, Jong-Min Lee, Mijin Park, Ji-Soo Kim, Marianne Dieterich

**Affiliations:** ^1^Department of Neurology, Jeonbuk National University Hospital, Jeonju-si, South Korea; ^2^Research Institute of Clinical Medicine of Jeonbuk National University, Jeonbuk National University Hospital, Jeonju-si, South Korea; ^3^Department of Biomedical Engineering, Hanyang University, Seoul, South Korea; ^4^Nuclear Medicine, School of Medicine, Jeonbuk National University Hospital, Jeonju-si, South Korea; ^5^Department of Neurology, Seoul National University Bundang Hospital, Seoul National University School of Medicine, Seoul, South Korea; ^6^Department of Neurology, Ludwig-Maximilians-University, Munich, Germany; ^7^German Center for Vertigo and Balance Disorders (IFB^*LMU*^), Ludwig-Maximilians-University, Munich, Germany; ^8^Munich Cluster for Systems Neurology (SyNergy), Munich, Germany

**Keywords:** Mal de Debarquement syndrome (MdDS), transient Mal de Debarquement (t-MdD), functional connectivity MRI, [18F]FDG PET, visuospatial memory, vestibular network, multisensory integration, emotional network

## Abstract

**Background:** Mal de debarquement syndrome (MdDS) is characterized by a subjective perception of self-motion after exposure to passive motion, mostly after sea travel. A transient form of MdDS (t-MdDS) is common in healthy individuals without pathophysiological certainty. In the present cross-sectional study, the possible neuropsychiatric and functional neuroimaging changes in local fishermen with t-MdDS were evaluated.

**Methods:** The present study included 28 fishermen from Buan County in South Korea; 15 (15/28, 53.6%) participants experienced t-MdDS for 1–6 h, and 13 were asymptomatic (13/28, 46.4%). Vestibular function tests were performed using video-oculography, the video head impulse test, and ocular and cervical vestibular-evoked myogenic potentials. Visuospatial function was also assessed by the Corsi block test. Brain imaging comprised structural MRI, resting-state functional MRI, and [18F]FDG PET scans.

**Results:** The results of vestibular function tests did not differ between the fishermen with and those without t-MdDS. However, participants with t-MdDS showed better performance in visuospatial memory function than those without t-MdDS (6.40 vs. 5.31, *p*-value = 0.016) as determined by the Corsi block test. Structural brain MRIs were normal in both groups. [18F]FDG PET showed a relative hypermetabolism in the bilateral occipital and prefrontal cortices and hypometabolism in the vestibulocerebellum (nodulus and uvula) in participants with t-MdDS compared to those without t-MdDS. Resting-state functional connectivities were significantly decreased between the vestibular regions of the flocculus, superior temporal gyrus, and parietal operculum and the visual association areas of the middle occipital gyrus, fusiform gyrus, and cuneus in participants with t-MdDS. Analysis of functional connectivity of the significant regions in the PET scans revealed decreased connectivity between the prefrontal cortex and visual processing areas in the t-MdDS group.

**Conclusion:** Increased visuospatial memory, altered metabolism in the prefrontal cortex, visual cognition cortices, and the vestibulocerebellum, and decreased functional connectivity between these two functional areas might indicate reductions in the integration of vestibular input and enhancement of visuospatial attention in subjects with t-MdDS. Current functional neuroimaging similarities from transient MdDS via chronic MdDS to functional dizziness and anxiety disorders suggest a shared mechanism of enhanced self-awareness as a kind of continuum or as overlap disorders.

## Introduction

Mal de Debarquement syndrome (MdDS) was recognized as a clinical entity for the first time in 1987 (1). The syndrome is characterized by a persistent rocking or swaying sensation occurring after prolonged passive movements, such as boat travel, that lasts for months or years, leading to balance problem, visuospatial and cognitive dysfunction (2). In contrast to typical MdDS, transient episodes of MdDS (t-MdDS) are fairly common even among healthy young individuals (3, 4). The post-motion-triggered rocking sensation is a common experience in healthy subjects with a prevalence of approximately 70% (4, 5) and is referred to “land-sickness.”

The underlying mechanism of MdDS and t-MdDS is not yet clear. Traditionally, MdDS has been considered a dynamic and multi-sensorimotor form of central nervous system adaptive plasticity with delayed or defective readaptation of the vestibular system after cessation of motion. According to the neural mismatch theory, this delayed readaptation leads to intersensory conflict (6). For example, passengers are exposed to a series of contradictory vestibular, visual, and proprioceptive stimuli when at sea, which may cause adaptation to specific ship motions. After landing, the newly acquired visuovestibular perception are no longer appropriate, causing Mal de Debarquement which lasts until proper readaptation is achieved (7). Recent studies indicated that readaptation of the velocity storage for VOR pathway, i.e., the adaptive processes associated with roll-while-rotating, could be a source of body oscillations in MdDS (8, 9). Treatment based on the readaptation of the VOR also has led to a substantial improvement in 70% of the subjects with MdDS (8). Memory of an internal representation of external passive movement (i.e., release of stored vestibular information from the hippocampus) may underlie a mechanism of MdDS (6). In a recent neuroimaging study of 20 MdDS patients, an association was observed between resting-state metabolic activity and functional connectivity between the entorhinal cortex and amygdala (10). Gray matter volume alterations were also found in the visual-vestibular processing areas and in a structure involving default mode, salience and central executive networks (11). Due to the similar history and overlapping clinical features, t-MdDS and MdDS may likely share underlying brain mechanisms. We hypothesized that the transient motion illusion in subjects with t-MdDS is reflected by changes in brain metabolism and functional connectivity involving areas that process spatial information, similar to persistent pathological MdDS. In the current study, neuropsychological and functional neuroimaging studies were evaluated using [18F]FDG PET and functional connectivity magnetic resonance imaging (fcMRI) to determine alterations in visual-vestibular networks in participants with t-MdDS.

## Subjects and Methods

### Subjects

A total of 28 fishermen living in Buan County in Korea were enrolled in this prospective study from May to June 2018. After a thorough clinical history focusing on vestibular disorders and MdDS symptoms had been performed, the individuals were divided into two groups: participants with t-MdDS and those without t-MdDS symptoms. No participant suffered from persistent MdDS for more than a month. The criteria for t-MdDS were as follows: (1) a perception of rocking and swaying after disembarking; (2) symptoms lasting ≤ 1 month; (3) no other causes of peripheral inner ear or central nervous system disorders after evaluation with appropriate neurotological testing.

All participants underwent neurological and neurotological evaluations, including video-oculography (VOG), video head impulse test (vHIT), cervical and ocular vestibular-evoked myogenic potentials (cVEMPs and oVEMPs, respectively), [18F]FDG PET/CT, structural MRI, and resting-state fcMRI. General cognitive function was assessed using the Korean Mini-Mental State Examination (K-MMSE). The Korean Beck Depression Inventory (K-BDI) and the Korean Beck Anxiety Inventory (K-BAI) were also carried out. In addition, the visuospatial function test, as part of the Wechsler Adult Intelligence Scale (WAIS)-IV, and the Corsi block test using the iPad's Path Span application were performed. Every evaluations were performed during the fishing ban period, i.e., symptom-free period.

All participants gave informed consent and received monetary compensation for participation. The study was approved by the Institutional Review Board at Jeonbuk National University Hospital (IRB No. 2017-09-022).

### Vestibular Testing

#### Video-Oculography (VOG) (12)

Eye movements and gaze stability were examined using three-dimensional VOG (3D-VOG, SMI, The Netherlands). Eye movements and the ability to hold a steady gaze were evaluated during attempted fixation of visual targets located centrally or eccentrically (±30° horizontally, ±20° vertically). Spontaneous and gaze-evoked nystagmus, vibration and head-shaking nystagmus, positional tests, horizontal saccades, and smooth pursuit eye movements were evaluated. Digitized data were analyzed using MATLAB® software.

#### Video Head Impulse Test (vHIT)

VHIT was performed using a video-oculography system (SLMED, Seoul, Korea). Participants were examined at a distance of 1 m from the target at eye level. To ensure the reliability of examination, the goggles were fastened to the head with an elastic band to minimize slippage. Participants were seated in a height-adjustable chair, which allowed the examiner to adjust the height of the subject's head for optimal examination. Participants were instructed to look at a point on the wall 1 m ahead. The examination was conducted by an experienced examiner and manually performed more than 20 times (head rotation 15–20°, duration 150–200 ms, peak velocity > 150°/s) on both sides of each plane. Normal vHIT was defined as having a gain of ≤ 2 standard deviation (SD) of the age-matched normal gain reference range and no fixation catch-up saccades.

#### VEMPs

To record cervical VEMPs, subjects were in the supine position and asked to hold their head up 30° above the floor and rotate it contralaterally to ensure contraction of the sternocleidomastoid muscles (11). An active surface EMG electrode placed over the belly of the ipsilateral sternocleidomastoid and a reference self-adhesive Ag/AgCl electrode on the incisura jugularis of the sternum were used for the recording.

For the recording of ocular VEMPs, the active electrode was located on the infraorbital margin 1 cm below the center of the contralateral lower eyelid and the reference electrode was placed 1 cm below the active electrode. During monaural sound stimulation, participants were asked to fix their gaze on the target located 25° above eye level. Unilateral 500 Hz, 5 ms air-conducted sound tone bursts with a calibrated 100 dB intensity were used. Amplified EMG potentials were bandpass filtered at 10–3,000 Hz and then the data were averaged from the stimulus onset to 50 ms.

### Neuropsychological Tests

All participants underwent the Visual Object and Space Perception Battery (VOSP) for visuospatial perception, the Corsi block test for visuospatial memory, and K-MMSE for general cognition.

#### VOSP

The VOSP battery consists of object perception and space perception tests. The spatial perception function was evaluated using dot count, position discrimination, number location, cube analysis, and the block design test. In addition, visuospatial memory was assessed using the Corsi block test.

***i) Dot count:*** Participants were asked to count how many black dots are on a white card. There were 10 cards. One point was bestowed for every correct count and maximum score is 10.
***ii) Position discrimination:*** Subjects were presented with 20 boards with two adjacent horizontal squares with a black dot (5 mm) at the center of each. One of the two squares had a dot in the center and the other was slightly off. The subject was asked to distinguish in which square the black dot was in the exact center. The examiner recorded the number of correct answers and maximum score is 20.
***iii) Number location:*** Subjects were presented with 10 boards with two adjacent vertical squares. The upper square had numbers arranged in a random and the lower square had only one black dot. The subject was asked to identify which number in the top square corresponded to the dot in the bottom square (maximum score: 10).
***iv) Cube analysis:*** Subjects were presented with 10 boards with three-dimensionally arranged cubes. Subjects were asked to identify how many cubes were on each board, including the hidden one (maximum score: 10). This test evaluated three-dimensional analysis presented on a two-dimensional plane.

#### Corsi Block Test

The examiner tapped cubes starting with a sequence of two blocks in front of the participant. Two trials were performed per block sequence length. The participant had to tap the cube sequence in the same order immediately after the examiner had finished. The number of cubes tapped ranged from 2 to 9. The subject had two chances to tap the cubes in the correct order; the subject only proceeded to the next step if he or she provided the correct answer.

### Psychometric Testing

#### Korean Beck Depression Inventory (K-BDI)

The Korean version of BDI-II is a 21-item self-report inventory which is designed to determine the presence and severity in depressive symptoms. Based on the severity in the last 2 weeks, each item is rated on a 4-point Likert-type scale ranging from 0 to 3.

#### Korean Beck Anxiety Inventory (K-BAI)

The BAI is also a self-report assessment of anxiety symptoms, which consists of 21 items rated on a 4-point Likert scale from 0 (not at all) to 3.The total score ranges from 0 to 63 in each test, with higher scores inferring more severe depressive (BDI) and anxiety (BAI) symptoms.

### Imaging Data Acquisition and Analysis

#### FDG PET

All participants fasted for at least 6 h prior to the intravenous injection of [18F]FDG, and blood glucose levels in all patients were < 126 mg/dL. Approximately 5.5 MBq of [18F]FDG per kilogram of body weight was administered intravenously. Scanning was performed approximately 60 min after [18F]FDG administration. Brain images were obtained using a Biograph TruePoint 40 PET/CT scanner (Siemens Medical Solutions, Knoxville, TN, USA). A CT scan was first obtained using a continuous spiral technique (120 kVp, 160 mA, 0.5 s rotation time). Next, a PET scan was taken in a three-dimensional mode for 10 min. The obtained PET data were iteratively reconstructed using an ordered-subset expectation maximization algorithm (128 × 128 matrix, 3.27 mm slice thickness, subset: 21, iterations: 2). The [18F]FDG PET/CT images were reviewed at a workstation (Syngo MI applications, Flexible Display 7.0.7.7; Siemens Medical Solutions, Erlangen, Germany).

#### Structural MRI and fMRI

Structural and functional images were acquired on a 3T MRI system (Magnetom Verio, Siemens Healthcare, Erlangen, Germany) with a 12-channel head coil. In a single session, 195 volumes (60 contiguous, axial, 2.5 mm-thick slices each; 1-mm gap) were acquired with a gradient echo, echo-planar imaging (EPI) T2^*^-sensitive sequence (repetition time: 2,000 ms; echo time: 30 ms; flip angle: 90°; matrix: 64 × 64; field of view: 192 × 192 mm). To reduce head movement and consequently artificial activation patterns, a foam pad was wrapped around the headphones. Anatomical images included a T1-weighted magnetization-prepared rapid gradient echo (MP-RAGE) sequence with a 256-mm field-of-view and 1.0 × 1.0 × 1.0 mm^3^ isotropic spatial resolution (TE, 4.37 ms; TR, 2,100 ms; 160 slices). Subjects were instructed to minimize movement and keep their eyes closed but not fall asleep.

Resting-state fMRI data were preprocessed with AFNI software (http://afni.nimh.nih.gov/) (13). After discarding the initial five volumes from each fMRI, images were de-spiked, slice timing was applied, and head motion was corrected. In the head motion correction, all functional scans were realigned to the first image with a 6-parameter, rigid body, spatial transformation, and differentiated head realignment parameters across frames yielded a six-dimensional time course representing instantaneous head motion. The anatomical image was co-registered to the functional image using affine registration with a Local Pearson Correlation cost function. The eroded white matter mask and eroded large ventricle (LV) mask were also transformed to EPI space. All images and masks in native space were normalized to a standard MNI 152 template and resampled with an isotropic voxel size of 2 mm. The normalized fMRI data were spatially smoothed with a 6-mm full width at half maximum (FWHM) Gaussian kernel (14). The normalized and smoothed fMRI data were corrected using a regression model with the nuisance signal removed with an anatomy-based correlation correction (ANATICOR) method (15). The anatomy-based regressors were extracted before spatial smoothing to avoid mixing signals from different tissues. The regressors of the ANATICOR method were as follows: (1) 6 parameters obtained by head motion correction, (2) the signal from the eroded LV mask, and (3) the signal from the eroded WM mask in the local neighborhood (*r* = 15 mm) of the voxel. The censoring was performed together in the nuisance regression model. The censoring was applied to fMRI data with the Euclidian norm of the first derivative of head motion > 0.25. The regressed and censored images were temporally bandpass-filtered (0.009 < f < 0.08) to reduce physiological noise.

#### Defining Regions of Interests (ROIs) and Seed-Based Functional Connectivity Analysis

The predefined established seed regions of known visual and vestibular processing areas were used to generate correlation maps (*p* < 0.05, FWE correction). The seeds included the bilateral posterior insula, inferior insula, superior temporal gyrus, parietal operculum, inferior parietal lobule (IPL), precuneus, thalamus, cerebellar flocculus and nodulus, vermis and vestibular nuclei in the brainstem. The structural boundaries of the whole seeds were delineated instead of defining spherical seeds within each region. The location of each seed was manually modified until it was within the boundary of each seed region in every subject. In order to analyze the functional connectivity of the significant regions in the PET analysis results, MNI coordinates of increased metabolic regions as well as widespread activation of the prefrontal cortex were used as follows: bilateral superior frontal cortex (SFC, x/y/z = 28/4/58 and −30/2/58), inferior prefrontal cortex (IPFC, x/y/z = 32/24/0 and −28/24/2), lateral orbital frontal cortex (LOFC, x/y/z = ±46, 34, 0) and bilateral dorsolateral prefrontal cortex (DLPFC, x/y/z = 30/46/20 and −34/52/14). These regions of interest (ROIs) were described as a 6-mm radius sphere at the peak using a voxel mask.

A region of interest (ROI)-based approach with *a priori* selected regions was used for resting-state functional connectivity analyses. For each seed, a resting-state time series was extracted separately for each subject by computing the mean BOLD intensity of all voxels within the seed boundary at each MR frame (time point). A correlation map of each seed was obtained *via* correlation analysis between the seed reference time series and the time series of the rest of the brain in a voxel-wise manner. The correlation map for each subject was converted to a z-value using Fisher's r-to-z transformation. The Mann-Whitney U-test was performed to compare functional connectivity between the subjects with and without t-MdDS. Monte Carlo simulation was conducted to control type I errors by calculating the significance level combination of cluster size and uncorrected individual voxel *p*-value (16). The simulation parameters were as follows: uncorrected individual voxel *p* = 0.02, simulation = 10,000 times, 8 mm FWHM Gaussian filter width with a whole brain mask. The Mann-Whitney U test was corrected by *P*α < 0.05 level (uncorrected individual voxel height threshold of *p* < 0.02 with a minimum cluster size of 306 voxels).

### Data Availability Statement

All of the individual participant data that underlie the results reported in this article, after deidentification (manuscript, tables, and figures) will be shared.

### Statistical Analysis

All metrics were compared between groups using the non-parametric Mann-Whitney *U*-test. Fisher's exact test was used for univariate comparison regarding sex and motion sickness. The Student's *t*-test was applied to test age and educational level as well as the Corsi block test. The Mann-Whitney *U*-test was used to compare the mean values of the two groups in MMSE, time at sea, and the visuospatial memory function test. A *p* < 0.05 was considered statistically significant. Analyses were performed using the Statistical Package for Social Sciences software (SPSS, Inc., Chicago, IL, USA).

## Results

### Demographic and Clinical Data

Twenty-eight fishermen who resided in Buan County participated in and completed the present study. Fifteen of the participants experienced a transient sense of rocking and swaying motion immediately after landing (t-MdDS, 15/28, 53.6%) and 13 participants did not (13/28, 46.4%). The demographic and clinical characteristics of the participants with t-MdDS and those without are summarized in Table 1. No differences in age, sex, educational level, K-MMSE score, and total time spent at sea were observed between the two groups. A female predominance in MdDS has been noted in many studies varying from 75% to >95% and age of onset is typically in the 40 to 50 s. In the current study, however, t-MdDS appears to be consistent in both men and women in the 50 s. Symptoms described shortly after landing included rocking and swaying and the mean duration was 3.27 h (range, 2–6 h). No participant in the t-MdDS group became seasick while at sea and similarly, motion sickness with other transportation was experienced less in participants with t-MdDS than in those without (Table 1).

**Table 1:** Demographic and clinical characteristics of participants with MdDS and those without t-MdDS.

**Characteristics**	**With t-MdDS (15/28, 53.6%)**	**Without t-MdDS (13/28, 46.4%)**	***p*-value**
Age	50.9 ± 6.0	56.7 ± 8.2	0.06^†^
Sex (male), *n* (%)	6 (40.0)	6 (46.2)	1.00^*^
Education (years)	10.2 ± 2.7	9.1 ± 2.7	0.30^†^
MMSE	28.8 ± 1.4	27.6 ± 1.7	0.06^**^
K-BDI^a^	22.7	15.5	0.11^†^
K-BAI^b^	7.92	8.8	0.39^†^
Handed (Right), *n* (%)	15 (100)	12 (100)	
**Time at sea**			
Total (years)	18.9 ± 7.7	20.1 ± 17.5	0.89^**^
Months per year (months)	7.6 ± 1.8	6.9 ± 1.7	0.13^**^
Hours per day (hours)	8.1 ± 2.1	6.7 ± 4.0	0.25^**^
**Duration of symptoms (hours)**			
Mean (SD)	3.27 (1.39)		
Median	3		
Range	2–6		
Motion sickness, n (%)	1 (6.7)	7 (58.3)	0.02^*^
Migraine	6 (40)	3 (23)	0.47
Aggravating factors	Bad sea conditions (windy with large waves)		
Relieving factors	Sufficient rest, lying down, or sleeping, during motion and driving		

* Fisher's exact test;

† Student's t-test;

** Mann-Whitney U-test;

a K-BDI, Korean Beck Depression Inventory;

b *K-BAI, Korean Beck Anxiety Inventory*.

### Vestibular Testing

Spontaneous or induced nystagmus was not observed in either group during VOG recordings. vHITs were normal for all six semicircular canals on both sides in all participants. There were no abnormalities in the cVEMP and oVEMP amplitudes and latencies in both groups.

### Neuropsychological and Psychometric Tests

Visuospatial perception and general cognition did not differ between groups with and without t-MdDS (Table 2). However, in the Corsi block test, a predominant method for assessing spatial memory capacity, participants with t-MdDS performed significantly better than those without symptoms (6.40 ± 0.91 vs. 5.31 ± 1.24, *p* = 0.016, Mann-Whitney test; Table 2).

**Table 2:** Results of visuospatial memory function tests in participants with and without t-MdDS.

	**With t-MdDS (*n* = 15)**	**Without t-MdDS (*n* = 13)**	***p*-value^a^**
**Visuospatial perception**			
Position discrimination	18.9 ± 1.80	18.4 ± 4.0	0.94
Number location	7.7 ± 3.2	6.5 ± 2.6	0.20
Cube analysis	9.3 ± 1.5	8.3 ± 2.1	0.07
Block design	8.9 ± 2.2	8.4 ± 3.5	0.79
**Visuospatial memory**			
Corsi block test	6.4 ± 0.9	5.3 ± 1.2	0.016

a *Mann-Whitney U-test*.

A comparison of the mean scores of the Korean version of BDI across two groups did not reveal significant differences between the t-MdDS group (mean = 22.69, SD = 28.9) and the group without t-MdDS (mean = 15.46, SD = 17.3, *p* = 0.11, Table 1). BAI also did not show differences between the two groups (mean = 7.92, SD = 3.67 vs. mean = 8.8, SD = 11.1, *p* = 0.39, Table 1). Correlation analysis with the BDI / BAI did not reveal any significant correlation in the brain structures with different metabolic activation or deactivation patterns.

### FDG-PET Analysis

**Figure 1:**
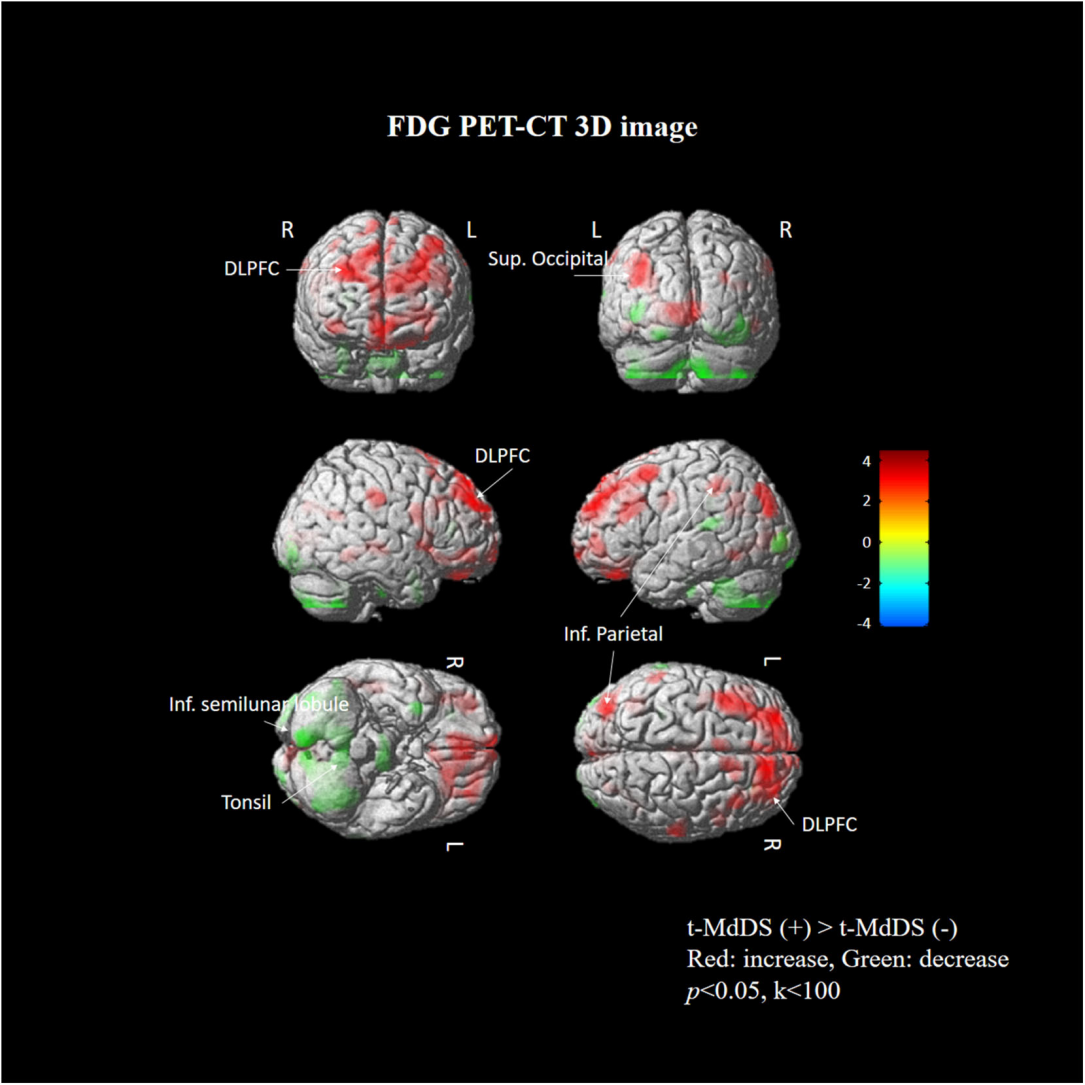
Areas of relative hyper- and hypometabolism in participants with t-MdDS compared with those without. Image is presented at *z* > 2.57 for better visualization; extent voxels: 0.

Subtraction analysis between groups with and without t-MdDS showed an increased metabolism in the left superior occipital, superior and inferior parietal lobules, and the right superior frontal gyrus including the dorsolateral prefrontal cortex (DLPFC) in subjects with t-MdDS compared to those without. In addition, hypometabolism was observed in t-MdDS participants bilaterally in the cerebellum including the right inferior semilunar lobule, nodulus, and the vermis especially the uvula (*p* < 0.05, uncorrected, Figure 1 and Table 3).

**Table 3:** Comparison of [18F]FDG PET/CT between participants with and without t-MdDS.

**Brain Regions**	**Side**	**Coordinates (mm)**			**Z-score**
		***x***	***y***	***z***	
**Increased**					
Superior occipital gyrus	L	−40	−84	34	2.74
Superior frontal gyrus	R	32	52	29	3.52
Superior parietal lobule	L	−38	−75	50	3.46
Inferior parietal lobule	L	−63	−39	44	2.81
**Decreased**					
Inferior semi-lunar lobule	R	10	−80	−38	−2.76
Nodule		10	−50	−28	−2.55
Uvula		−4	−81	−35	−2.52
Tonsil	L	−34	−58	−39	−2.38

### Structural MRI

Volume changes in regional gray matter were not detected at a threshold of *p* < 0.001 uncorrected for the whole brain between groups with and without t-MdDS.

### Resting-State fMRI

**Figure 2:**
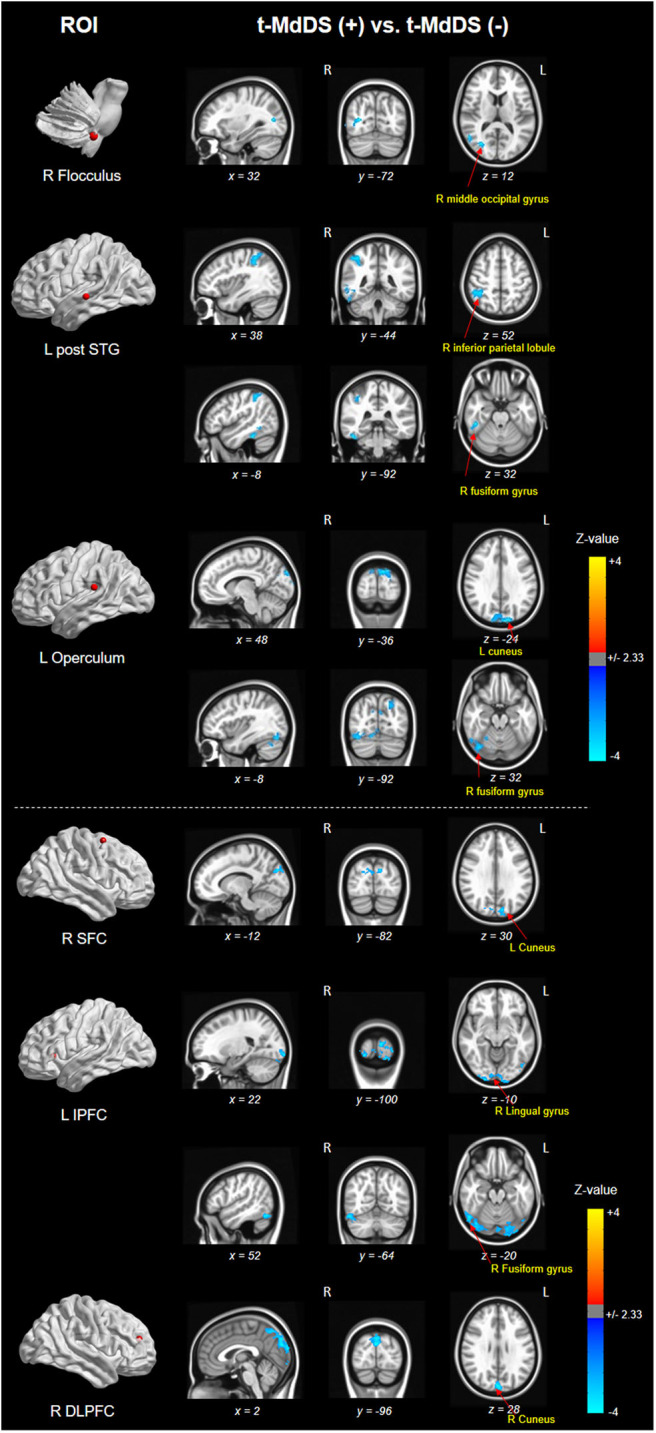
Results of functional connectivity for three ROIs of the vestibular processing regions in both groups. Negative (cold color) *z*-value indicates that functional connectivity of subjects with t-MdDS is significantly lower than functional connectivity of subjects without symptoms (*P*α < 0.05).

Different resting-state functional connectivities were observed at three ROIs (right flocculus, left posterior superior temporal gyrus, and left parietal operculum OP2) of the vestibular processing regions (Figure 2 and Table 4, Mann-Whitney *U* test). Compared with participants without t-MdDS, subjects with t-MdDS showed decreased functional connectivity between the right flocculus and right middle occipital gyrus (*z*-value: −3.757, *P*α < 0.05), between the left posterior superior temporal gyrus and right inferior parietal lobule (*z*-value: −3.66, *P*α < 0.05) and the right fusiform gyrus (*z*-value: −3.757, *P*α < 0.05), and between the left parietal operculum and left cuneus (*z*-value: −4.233. *P*α < 0.05) and right fusiform gyrus (*z*-value: −3.611, *P*α < 0.05). The inferior and posterior insula, which are considered core structures of vestibular processing and integration, did not differ in the resting-state functional connectivities between both groups with and without t-MdDS.

**Table 4:** Different resting-state functional connectivities between participants with and without t-MdDS.

**Seeds**	**Brain regions**	**Side**	**Coordinates (mm)**			**Minimum**	**Voxels**
			***x***	***y***	***z***	**z**	
Flocculus, R	Middle occipital gyrus	R	32	−72	12	−3.757	426
Posterior superior temporal gyrus, L	Inferior parietal lobule fusiform gyrus	R R	38 48	−44 −36	52 −24	−3.66 −3.757	403 332
Parietal operculum 2, L	Cuneus fusiform gyrus	L R	−8 38	−92 −74	32 −20	−4.233 −3.611	929 657
Superior FC, R	Cuneus	L	−12	−82	30	−3.708	307
Inferior PFC, L	Lingual gyrus	R	22	−100	−10	−4.198	2160
	Fusiform gyrus	R	52	−64	−20	−4.031	609
DLPFC, R	Cuneus	R	2	−86	28	−3.939	975

*FC, frontal cortex; PFC, prefrontal cortex; DLPFC, dorsolateral prefrontal cortex; R, right; L, left*.

**Figure 3:**
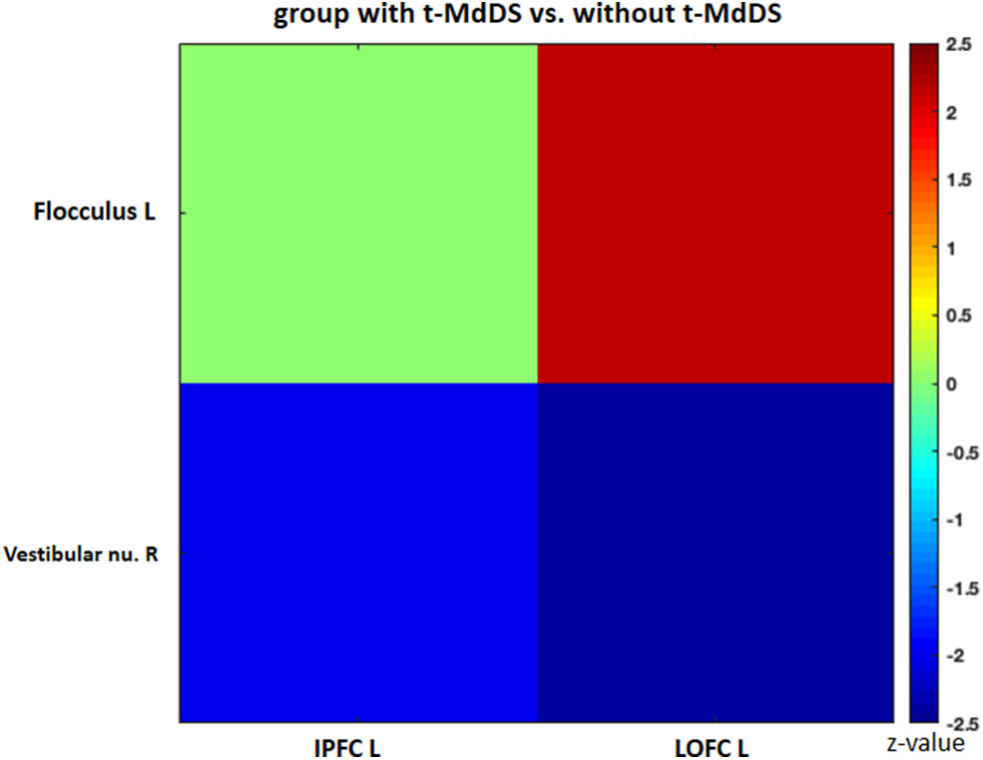
Functional connectivity of the significant regions from the FDG-PET analysis. IPFC, inferior prefrontal cortex; LOPFC, lateral orbital prefrontal cortex; R, right; L, left.

Sub-analysis of functional connectivity for the significant regions in the PET analysis, including widespread activation of the prefrontal cortex, revealed that the t-MdDS group showed decreased functional connectivity between the right superior frontal cortex and left cuneus, between the left inferior prefrontal cortex and right lingual and fusiform gyri, and between the right DLPFC and right cuneus compared to participants without t-MdDS (Table 4). Functional connectivity between the prefrontal cortex and the vestibular processing regions was increased between the left lateral orbital PFC and the left flocculus; it was decreased between the right vestibular nuclei and left inferior PFC and lateral orbital PFC (Figure 3).

## Discussion

Task-free resting-state fcMRI and FDG PET combined with neuropsychological tests were used in the present study to investigate the functional brain connectivity and metabolic signatures of t-MdDS in local fishermen. Visuospatial memory function was significantly higher, and changes in brain glucose metabolism and functional connectivity in the vestibular and visuospatial attention processing areas were observed in the t-MdDS group compared with the group without t-MdDS. FDG metabolism in t-MdDS participants was significantly increased in several regions, especially widespread bilaterally in the DLPFC and OFC that is involved in integration of different cognitive operations as well as in emotions. The prefrontal cortex plays a vital role in mood-regulating circuits, anxiety disorders, and fast responses to threats (17) and the OFC is involved in sensory integration and in representing the affective value and expectation (18). Metabolism increases were also seen in visual areas including the superior occipital gyrus responsive to visual orientation and to visuospatial attention. Further, an increase was found in the superior and inferior parietal lobules, which also contribute to spatial attention and reorientation (Figure 1 and Table 3). This increased metabolism in the areas associated with visuospatial attention and orientation might reflect increased visuospatial memory function determined with the Corsi block test.

In addition, glucose metabolism was significantly reduced in the vestibulocerebellum, including the nodulus and uvula, in t-MdDS participants. This could indicate an adaptive mechanism that suppresses the enhanced visual-vestibular inputs during continuous movement on a boat. Alternatively, decreased function in the vestibulocerebellum may cause inadequate suppression of enhanced visuospatial memory induced by continuous oscillation of the visual environment. In combination with the bilateral enhanced metabolism of the prefrontal cortex, the reduced cerebellar metabolism may more likely represent a reduction of automatic control, since the cerebellum is important for several aspects of sensorimotor integration such as subconscious automatic motor control (19).

**Figure 4:**
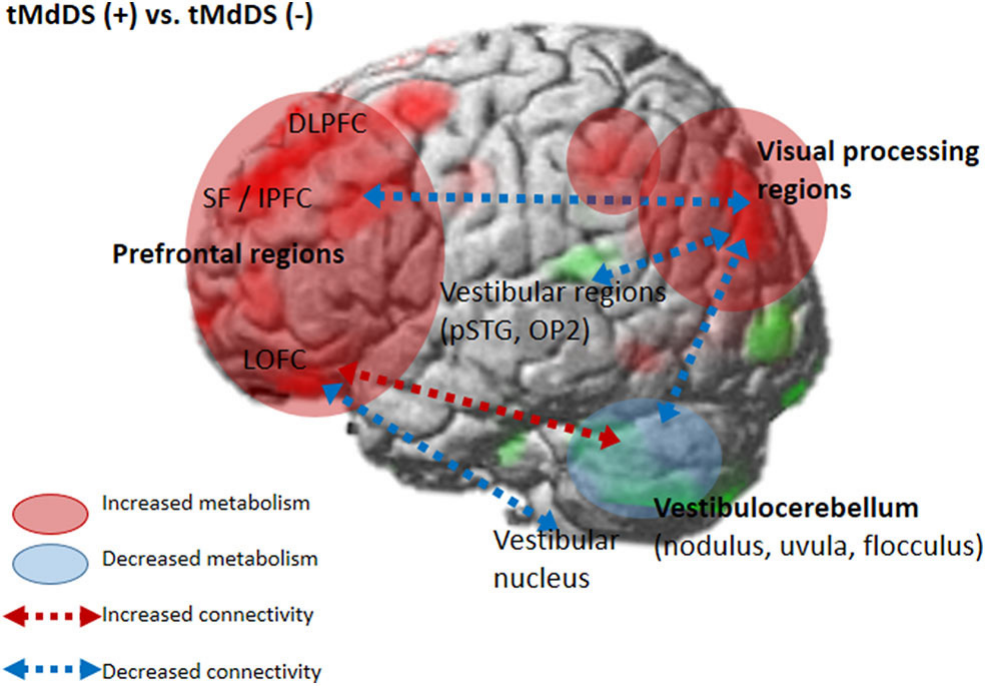
Brain metabolic and functional connectivity changes in the t-MdDS group. Schematic descriptions are superimposed on the cortical [18F]FDG-PET results.

We further assessed functional connectivity changes between the visual and vestibular sensory processing regions in the participants with t-MdDS compared to those without. During rest, fcMRI of the normal brain shows large-amplitude spontaneous low-frequency (<0.1 Hz) fluctuations that are temporally correlated across functionally related areas referred to as “resting-state functional connectivity” (rs-fc) (20). Connectivity between the vestibular regions and the visual association areas of the middle occipital gyrus, fusiform gyrus, and cuneus was significantly decreased in t-MdDS subjects based on rs-fc between the vestibular seed regions and whole-brain analysis (Figure 2 and Table 4). Sub-analysis for fc of the significant regions in the PET analysis results showed decreased connectivities between the prefrontal cortices and visual processing areas of the cuneus as well as lingual and fusiform gyri in the t-MdDS group (Figure 2 and Table 4). This could mean that the participants rely less on vestibular but more on visual input, i.e., they show a sensory shift from the vestibular to the visual system. Thus, the pattern of reduced connectivity between vestibular regions and visual cortex areas, reduced metabolism in the vestibulocerebellum, and increased metabolism in prefrontal and visual cortex areas reflects an enhanced conscious control of sensorimotor function under the lead of the visual system instead of an unconscious automatic control of stance and gait (Figure 4). A similar pattern was found in patients with functional dizziness, i.e., phobic postural vertigo (21), who are known to show a continuous co-contraction of antigravity muscles during normal stance that normalizes during distracting attention by dual task conditions (22). This co-contraction may be an expression of an irrational fear of imbalance which is observed also in specific phobias (23).

Clinically, the fishermen tended to have t-MdDS symptoms when they were exposed to a significant amount of motion in a boat on the sea during extreme weather with high winds and unpredictable large waves rather than when they spent a longer time on a calm sea (Table 1). Boat stability depends not only on the size of the boat but also on wind and wave conditions as well as the direction of the boat. Since fishing boats are usually small, substantial roll can occur depending on the size of the waves despite the ship's roll stabilizers. Modulation of roll motion mostly depends on the integrity of the cerebellar nodulus (24–26). In animal and human experiments, the nodulus and uvula exert powerful control on the velocity storage integrator. The lateral portions of the nodulus cause discharge activity in velocity storage during visual suppression as well as loss of stored activity in velocity storage during tilt-suppression (27–30). In addition, the central parts of the nodulus generate activities responsible for orienting the axis of eye, head, and body velocity to the spatial vertical (26, 28, 30, 31), and habituation of the time constant of the VOR (32). The majority of vestibular fibers in Scarpa's ganglion project directly to the Purkinje cells in the contralateral nodulus through the inferior olives that sense active or passive movements of the head and body (33). Thus, there is a strong projection to the nodulus continuously modulating the active or passive head and body movements. Vestibular neurons sense the (roll) position of the head and body and transmit this activity to the nodulus via the inferior olives. The nodulus also receives input from the inferior olive that originates in the nucleus of the optic tract which carries optokinetic-generated activity to the vestibular nuclei and the nodulus. Recently, the existence of visually driven Purkinje cells was revealed in the anterior part of the nodulus and ventral uvula near the midline (34). Studies of neural activity in the flocculus of alert monkeys showed that the main mossy fiber input to the flocculus originates in the vestibular nuclei and a second input of unknown origin conveys visual information from retinal slip (35). Thus, part of the flocculus may also be specialized to work visual-vestibular interactions. Convergence of vestibular and visual motion information is essential for accurate spatial orientation and navigation. Therefore, prolonged exposure to vestibular and moving visual inputs, when at sea, could condition the motion-related neurons or visually driven Purkinje cells in the nodulus and flocculus, subsequently contributing to changes or even maladaptation of the velocity storage integrator or visual-vestibular interaction in the vestibulocerebellum.

Another interesting finding was that participants in the t-MdDS group did not become seasick while at sea and experienced less motion sickness with other methods of transportation than those without t-MdDS (Table 1). The mechanism of this relative tolerance of motion sickness in the t-MdDS group remains unclear, but the properties of visuovestibular interaction also could be explained. As our data showed that the t-MdDS participants depend more on the visual than the vestibular system in the setting of consistently spontaneous motion, the participants may become more tolerant of slow changes in the visual surroundings because the visual system is usually used to stabilize low-frequency postural and visual sway. Indeed, a low-frequency oscillatory motion stimulation of 0.1 to 0.4 Hz as a roll and pitch rotation was found to be a typical experimental stimulus for t-MdDS in humans, especially when it was more unpredictable and along multiple dimensions (36). These low frequencies include both the frequency of natural stimuli n the boat (37) as well as the predominant frequency of 0.2–0.3 Hz of rocking sensations and body oscillations in individuals with MdDS (8, 9). During continuous activity within the visual system on a boat, reciprocal inhibitory activity of the visual and vestibular systems (38) leads to a suppression of vestibular processing areas in order to reduce the visuovestibular functional conflict. This tolerance to motion sickness with other transportation in subjects with t-MdDS is also consistent with Dai's readaptation hypotheses, which implies that if velocity storage path of the VOR cannot adapt, subjects experience motion sickness, and if VOR does adapt, then the subject may get MdDS (8).

Perception of motion and space information in the brain is processed in a widely distributed network involving the vestibular, visual, and somatosensory cortices because the interactions among these sensory systems are important for postural and spatial perception. In contrast to other sensory modalities of visual or auditory inputs, a unimodal primary sensory cortex for vestibular inputs does not exist. Instead, a multimodal vestibular cortical network contains neurons integrating information from vestibular, visual, auditory, and somatosensory stimuli. These multimodal sensory integrating neurons have been found not only in the vestibular brainstem nuclei but also in particular in several cortical regions centered around the posterior insula, retroinsular region, parietal operculum, and adjacent posterior perisylvian regions of the parietal and temporal cortices which superimpose the vestibular cortex and contribute to perception of spatial cognition and gravity (39, 40). Perception of self-motion is processed by the multimodal nature of the vestibular information in which visual signals as well as somatosensory and auditory inputs collectively provide information (41). However, detection of movement in space may often be more dependent on visual input than vestibular and somatosensory information, since it is necessary to stabilize the eyes on targets of interest. Significantly increased glucose metabolism in the visuospatial attention regions with reduced metabolism in the vestibulocerebellum and decreased resting-state functional connectivity between these two functional regions in the participants with t-MdDS indicate reductions in the integration of vestibular input and enhancement of visuospatial attention. The wide-spread increased glucose metabolism in the prefrontal and frontal cortex bilaterally and the visual processing areas associated with decreased functional connectivity between them also raise the question of whether these changes within the vestibular and visual networks could be due to an enhanced self-awareness, self-attention, and emotional processes (Figure 4). Increased functional connectivities were also found between the vestibulocerebellum (flocculus) and the orbitofrontal cortex in t-MdDS participants, i.e., within brain networks important for adaptive and goal-directed behavior (Figure 3). Similarly altered connectivities within the prefrontal and orbitofrontal cortex have been observed in patients with mood disorders (42, 43). The enhanced connectivity of these networks combined with decreased fc between the visual, vestibular and prefrontal networks in our study might explain the features of over-generalization and anxious response to certain stimuli, a disturbed self-awareness, and an overacted compensatory mechanism for evaluating the specific stimuli in the participants with t-MdDS (21). The dorsolateral prefrontal cortex influences multiple interconnected networks with effects on mood, cognition, and visuospatial processing (44–47). As mentioned above, similar patterns of increased metabolism or connectivity in the prefrontal cortex combined with a decrease in the vestibulocerebellum were also found in patients with functional dizziness (21). The patients with functional dizziness also shift their attention from the vestibular to the visual system. Based on such similarities, an overlap in a kind of continuum from transient MdDS via chronic MdDS to functional dizziness and anxiety disorders, all with enhanced self-awareness, can be suggested. Therefore, these networks may process the data differently under normal conditions such as when t-MdDS enhanced attention to the visual system and to more conscious balance control.

## Data Availability Statement

The raw data supporting the conclusions of this article will be made available by the authors, without undue reservation.

## Ethics Statement

The studies involving human participants were reviewed and approved by the Institutional Review Board at Jeonbuk National University Hospital (IRB No. 2017-09-022). The patients/participants provided their written informed consent to participate in this study.

## Author Contributions

S-YO, J-SK, and MD contributed to the design and implementation of the research. S-HJ and J-JK contributed to gather and analyze the data and to the writing of the manuscript. Y-HP, Y-HH, H-JJ, J-ML, and MP contributed to the analysis of the imaging results and to the writing of the manuscript. All authors contributed to the article and approved the submitted version.

## Conflict of Interest

The authors declare that the research was conducted in the absence of any commercial or financial relationships that could be construed as a potential conflict of interest.
